# Localizations of visual cycle components in retinal pigment epithelium

**Published:** 2009-01-26

**Authors:** Jing Huang, Daniel E. Possin, John C. Saari

**Affiliations:** 1 Department of Ophthalmology, University of Washington, School of Medicine, Seattle, WA; 2 Department of Biochemistry, University of Washington, School of Medicine, Seattle, WA

## Abstract

**Purpose:**

We used immunocytochemistry and confocal microscopy to determine whether enzymes of the rod visual cycle were uniformly distributed in retinal pigment epithelium (RPE) cells. The localizations of these enzymes were compared to known localizations of retinoid-binding proteins and associated proteins.

**Methods:**

Antibodies to proteins and enzymes associated with the rod visual cycle were used for fluorescence immunocytochemistry with frozen sections of albino mouse and rat retina. Images were obtained with a laser scanning confocal microscope.

**Results:**

Components associated with the rod visual cycle were distributed in three distinct patterns in mouse and rat RPE. Three visual cycle enzymes (RDH5, LRAT, and RPE65) were restricted to the somata of RPE cells and were not detected within apical processes. Ezrin, an actin-binding protein, and ERM-binding phosphoprotein50/sodium-hydrogen exchanger regulatory factor1 (EBP50/NHERF1), an ezrin-binding PDZ-domain protein, were largely restricted to RPE apical processes. The fluorescence intensity over Müller cell apical processes was less intense. Cellular retinaldehyde-binding protein (CRALBP), which binds to EBP50/NHERF1, and cellular retinol-binding protein type 1 (CRBP1) were found throughout RPE cells and Müller cells.

**Conclusions:**

Visual cycle enzymes were confined to the somata of RPE cells and did not occur within the long apical processes, either in dark- or light-adapted animals. Other components previously linked to the visual cycle (EBP50/NHERF1 and ezrin) were largely confined to the apical processes, where they could be associated with release of 11-*cis*-retinal or uptake of all-*trans*-retinol. CRALBP and CRBP1 were distributed throughout the RPE cell, where they could mediate diffusion of retinoids between apical processes and somata.

## Introduction

Regeneration of rod visual pigments occurs via two sequential reaction pathways, one in rod photoreceptor cells and one in retinal pigment epithelial (RPE) cells, which are coupled into a cycle by transport of retinoid substrates across the extracellular compartment separating the two cell types. Within rod cells, photo-isomerization converts 11-*cis*-retinal of rhodopsin to all-*trans*-retinal, which is then reduced to all-*trans*-retinol by NADPH. All-*trans*-retinol diffuses across the interphotoreceptor matrix (IPM) and enters the RPE cells. Within RPE cells, all-*trans*-retinol is esterified with a fatty acid and the resultant all-*trans*-retinyl ester is isomerized to 11-*cis*-retinol with hydrolysis of the ester bond. 11-*cis*-retinol is subsequently oxidized by NAD(P) to 11-*cis*-retinal, which leaves the RPE apical surface, diffuses to photoreceptor cell outer segments, and binds to opsin to regenerate rhodopsin. Several reviews have described the chemistry and enzymology of the visual cycle in detail [1-8].

Esterification of all-*trans*-retinol in RPE is due primarily to lecithin:retinol acyltransferase (LRAT), with minor contributions from acylCoA:retinol acyltransferase (ARAT) [9-12]. Isomerization and simultaneous hydrolysis of all-*trans*-retinyl ester to 11-*cis*-retinol can be attributed to the activity of RPE65 (isomerohydrolase) [13-15]. RDH5 plays a major role in the oxidation of 11-*cis*-retinol to 11-*cis*-retinal based on the delayed dark adaptation displayed by humans with mutations in the gene for this enzyme [16]. In *Rdh5*^−/−^ mice, the rate of rhodopsin regeneration is normal [17]; however, 13-*cis*-retinoids accumulate, indicating that visual cycle metabolites are not metabolized normally.

*Rdh11^−/−^* is another candidate for this activity in mice, but some results suggest it plays a minor role [18]. The dehydrogenase(s) responsible for reduction of all-*trans*-retinal in rod photoreceptor cells of mice has not been identified. Targeted disruption of individual candidate genes failed to have a major effect on rhodopsin regeneration rates [19,20]. 11-*cis*-retinyl ester hydrolase (REH) activity has been detected in RPE extracts but not yet assigned to a molecular entity.

Three water-soluble retinoid-binding proteins have been demonstrated to play roles in the rod visual cycle. CRALBP binds *cis*-retinoids with high affinity. Delays in dark adaptation resulted from mutations of its gene in humans (*RLBP1*) [21] and from disruption of its gene (*Rlbp1*) in mice [22]. Analysis of visual cycle retinoids in *Rlbp1^−/−^* mice retinas localized the impairment in the visual cycle to the isomerase reaction [22], which reinforced the proposed role for CRALBP as an acceptor for 11-*cis*-retinol from the isomerohydrolase [23,24]. Mutations in the *CRBP1* gene have not been associated with retinal diseases. Mice deficient in this protein have reduced amounts of hepatic and RPE retinyl esters [25,26], consistent with the role proposed for this protein from in vitro studies as a carrier of all-*trans*-retinol for enzymatic esterification by LRAT [27-30]. Interphotoreceptor retinoid-binding protein (IRBP) is present in the interphotoreceptor matrix, where it can solubilize and protect retinoids. No mutations have been reported in the *IRBP* gene in humans. *Irbp*^−/−^ mice have been shown to display slow photoreceptor degeneration [31]. However, the rate of rhodopsin regeneration was normal [32,33].

In addition to the known enzymes and retinoid-binding proteins of RPE, two scaffold proteins are associated with the visual cycle because of their interactions with CRALBP [34,35]. EBP50/NHERF1 is a scaffold protein with two Post-synaptic density 95/Discs Large/Zonula Occludens 1 (PDZ) domains and a C-terminal domain that binds ezrin [36,37]. At least one of these PDZ domains binds CRALBP via a conserved C-terminal PDZ-motif [34,35]. Ezrin is an indirect interaction partner of CRALBP by virtue of its affinity for a C-terminal domain of EBP50/NHERF1. Ezrin, in turn, can bind to F-actin via a C-terminal domain [36].

The RPE consists of a single layer of hexagonal cells that exhibit a striking polarization of plasma membrane components. As in other epithelial cells, regional specializations of cellular structure reflect the different roles of the apical, lateral, and basal surfaces of the cells. Basal RPE surfaces are characterized by pronounced infoldings, which abut the basement membrane separating the cells from the choroidal blood supply. Presumably this specialization reflects the need to maximize surface area for uptake of nutrients from and delivery of wastes to choroidal blood. Proteins of tight junctions, such as occludin and claudin, characterize the lateral surfaces of RPE cells. Apical surfaces of RPE cells elaborate long processes (approximately 20 μm), which extend to envelope outer segments of adjacent photoreceptor cells. This intimate association of RPE apical processes and photoreceptor outer segments has long been associated with nutritional exchange between these two cell types, including retinoid exchange during regeneration of visual pigment as described in the previous section. Regions of the apical surface are also involved in phagocytosis of shed portions of distal outer segments in the process of outer segment membrane renewal. Many proteins show a restricted distribution to one or another of these plasma membrane surfaces or regions of the cell [38,39].

Several enzymes of the rod visual cycle have been localized to RPE cells, but their regional distributions within these cells have not been examined in the context of rod visual cycle function. In addition, pigmented animals or older animals with lipofuscin accumulations were used for many of the localizations. The presence of pigment granules within the somata can mask or diminish fluorescence in this area, whereas the bright yellow fluorescence of lipofuscin can obscure weaker fluorescent signals. Here, we demonstrate that three enzymes of the visual cycle (LRAT, RPE65, and RDH5) are confined to somata of rodent RPE cells and restricted from the apical processes. This localization, when taken with the known pan-retinal localization of the retinoid-binding proteins and the predominantly apical localization of associated proteins, is likely to be important is generating the directed flows of retinoids characteristic of the visual cycle.

## Methods

### Animals

This study used 2-month-old female albino BALB/c mice and 10-week-old female albino Sprague Dawley rats. Mice were obtained from The Jackson Laboratories, Bar Harbor, ME, and rats from Charles River Laboratories, Wilmington, MA. Rats and mice were housed in appropriate rodent cages with 12 h light-12 h dark cycle unless specified otherwise, and provided with water and normal laboratory chow ad lib. All animal procedures were approved by the University of Washington Animal Care Committee and were performed in accord with the ARVO Statement for the Use of Animals in Ophthalmic and Vision Research.

### Sources of primary antibodies and fluorescent probes

Monoclonal anti-CRALBP (mAb B2) was generated with his-tagged human CRALBP as the antigen. The antibody recognized a single polypeptide of ~36 kDa by immunoblot and RPE and Müller cells by immunocytochemistry (ICC; Nawrot et al., 2004). Polyclonal (pAb) anti-CRALBP (pAb UW55) was produced in rabbits immunized with his-tagged human CRALBP. The antibody recognized a single polypeptide of ~36 kDa by immunoblots and RPE and Müller cells by ICC [22,34,35]. pAb anti-CRALBPs (pAbs Ig83, Ig84) were our original antibodies produced in rabbits immunized with purified bovine CRALBP. The antibodies recognized a single polypeptide of 36 kDa by immunoblots and RPE and Müller cells by ICC [40]. mAb anti-CRBP1 (mAb F3) was generated in house following a procedure we used earlier for preparation of anti-CRABP [41]. In brief, bovine CRBP was purified to apparent homogeneity, oxidized with performic acid, and used to immunize mice exactly as described [41]. Hybridoma cell lines were generated and selected based partially on their lack of cross reactivity with other closely related retinoid-binding protein (CRABP, CRALBP, IRBP). mAb F3 recognized a single polypeptide of ~16 kDa by immunoblot and RPE and Müller cells by ICC (this study). pAB anti-RPE65 (pAb PETLET) was a gift of T. Michael Redmond, National Eye Institute, Bethesda, MD. The antibody recognized a single polypeptide of ~65 kDa by immunoblot and RPE cells by ICC. mAb anti-RPE65 (mAb 9–22) was prepared by immunization of mice with microsomes from bovine RPE. Hybridoma cell lines were selected based on their recognition of 65 kDa protein in bovine RPE microsomes. The procedures used for mAb production were described earlier [41]. The antibody recognized a single polypeptide of ~65 kDa by immunoblot of bovine RPE and RPE cells in bovine and primate retina by ICC. mAb-9–22 did not recognize mouse RPE65 (data not shown). mAb anti-RDH5 (mAb D9) was produced by immunization of mice with his-tagged RDH5 [42]. The antibody recognized a single polypeptide of ~32 kDa by immunoblot and RPE cells by ICC42. mAb anti-LRAT (mAb B1) was produced by immunization of mice with a his-tagged fragment (Q89-E179) of mouse LRAT43. The antibody recognized, polypeptides of 32 and 25 kDa by immunoblot, both of which were absent in Lrat^−/−^ mice [43]. Anti-RDH5 and anti-LRAT were used with permission of Krzysztof Palczewski, Case Western Reserve, Cleveland, OH. pAB anti-ezrin (pAb B64) and pAB anti-EBP50 (pAb B62) were gifts of Anthony Bretscher, Cornell University, Ithaca, NY. PAB anti-calreticulin was purchased from Abcam, Inc. (Cambridge, MA; Abcam ab13504). Alexa Fluor® 488-phalloidin (F-phalloidin) and secondary antibodies labeled with Alexa Fluor® 568 (red) or Alexa Fluor® 488 (green) were obtained from Molecular Probes/Invitrogen, Carlsbad, CA.

### Imaging

**Figure 1:**
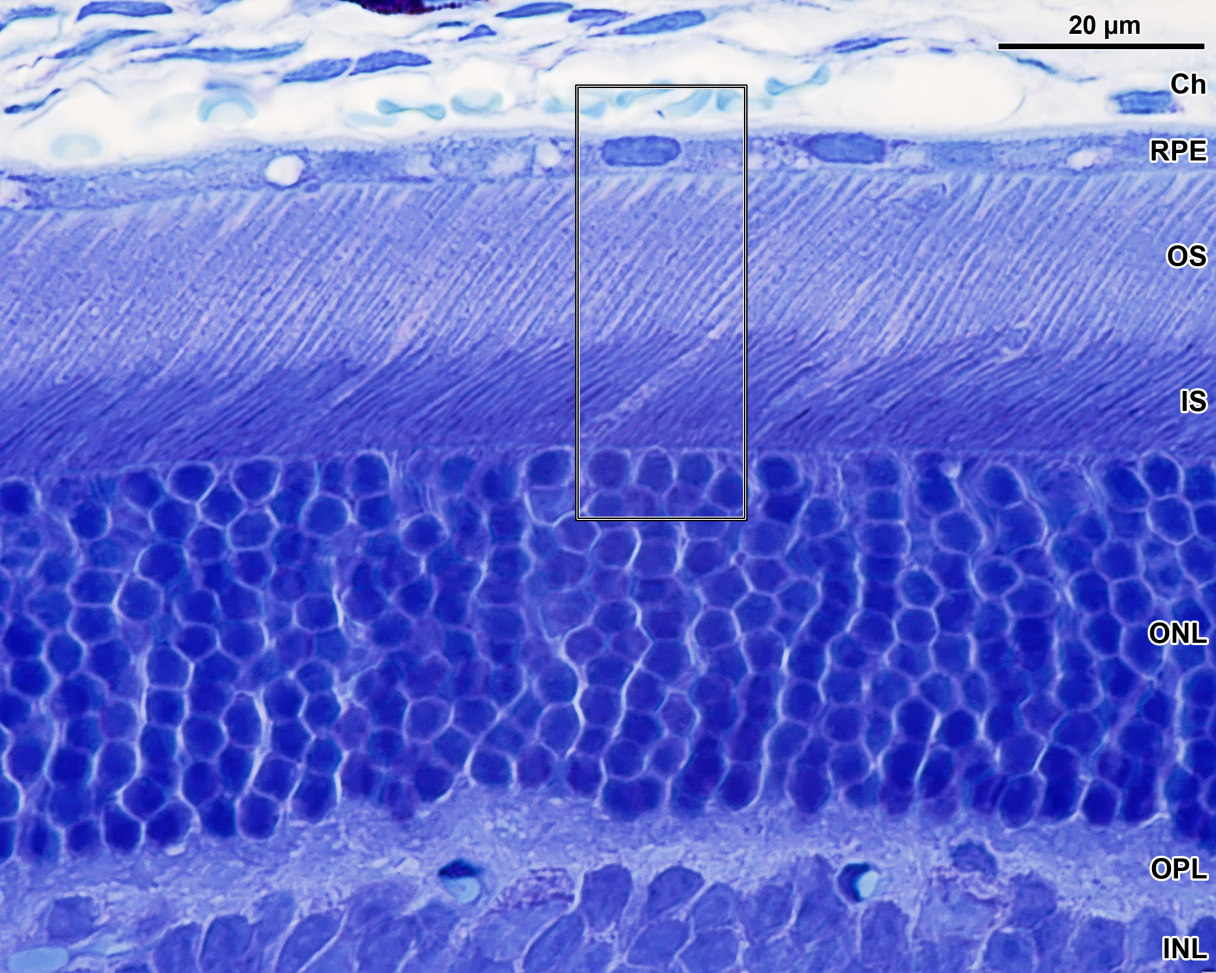
The interface of RPE and photoreceptor cells in albino mouse retina. The boxed area represents the approximate region of outer retina shown in subsequent immunocytochemistry images. Abbreviations: choroid (Ch); retinal pigment epithelium (RPE); photoreceptor cell outer segments (OS); photoreceptor cell inner segments (IS); outer nuclear layer (ONL); outer plexiform layer (OPL); inner nuclear layer (INL).

**Figure 2:**
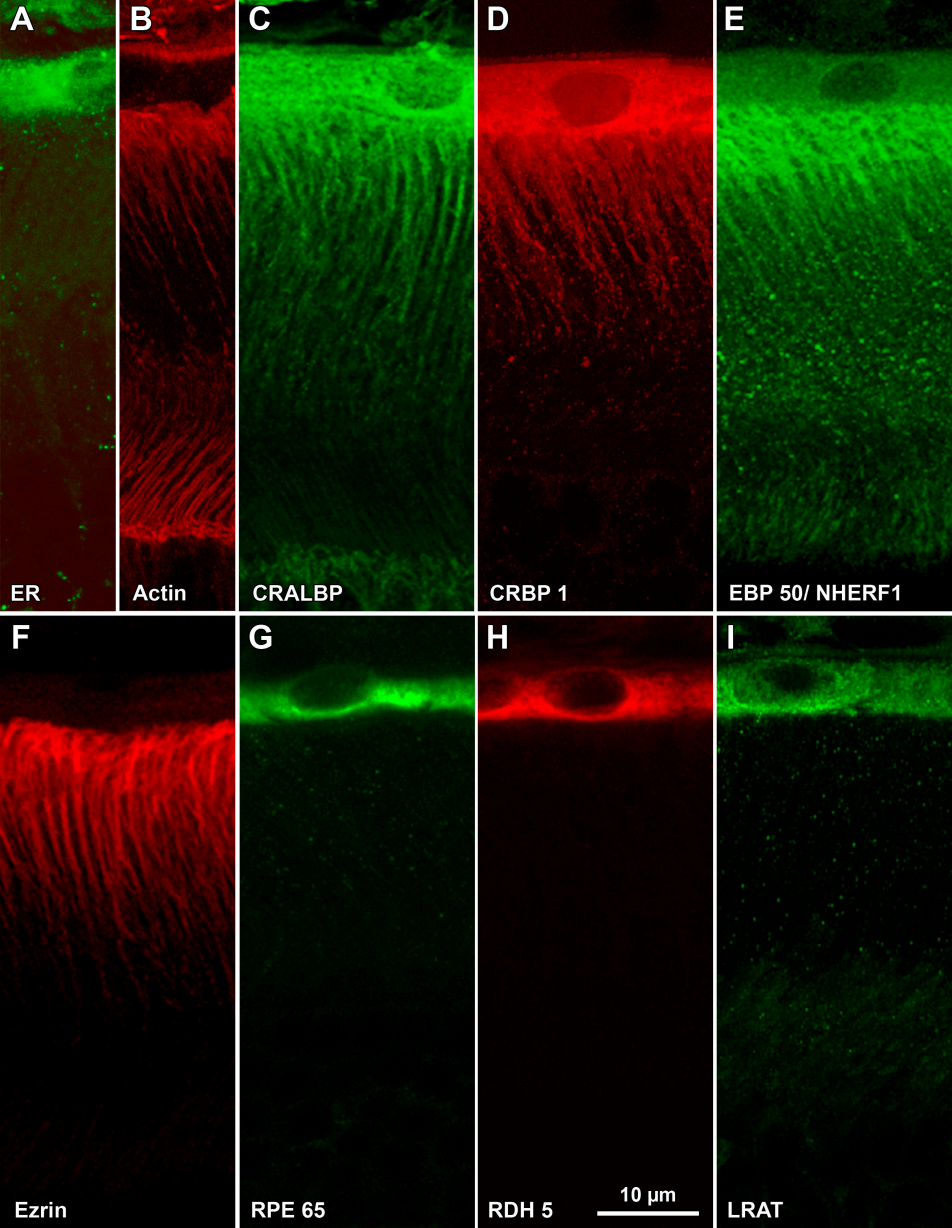
Immunostaining of albino mouse outer retina. Sections were labeled with fluorescent probes and analyzed by laser scanning confocal microscopy. The region of outer retina in these images is shown in Figure 1 **A:** Calreticulin, a marker for the endoplasmic reticulum, is found within the RPE cell body but not its apical processes. **B:** Actin, stained with Alexa Fluor® 488-phalloidin, is found near the basal side of RPE, within RPE and Müller cell apical processes, and at the external limiting membrane. **C:** CRALBP is found within the entire RPE cell, including the apical processes. Nuclei appear to be labeled in this image; however, this was not a consistent feature of the images and is likely a Z-projection artifact. Distal tips of Muller cells, including their apical processes, are also labeled (pAb UW55). **D:** CRBP1 distribution in RPE cells is similar to that of CRALBP (mAb F3). E. EBP50/NHERF1 is found within RPE and Müller cell apical processes and, to a lesser extent, within the RPE cell body. The punctate nature of the signal has been a constant feature of our images (pAb B64). **F:** Ezrin is confined to RPE apical processes (mAb B62). **G:** RPE65 is found within the cell bodies of RPE cells (pAb PETLET). Apical processes are not labeled. **H:** RDH5 distribution is similar to that of RPE65 (mAb D9). **I:** LRAT distribution is similar to those of RPE65 and RDH5 (mAb B1). Images shown are Z-projections created from LSCM image stacks (Z_t_=3 μm).

**Figure 3:**
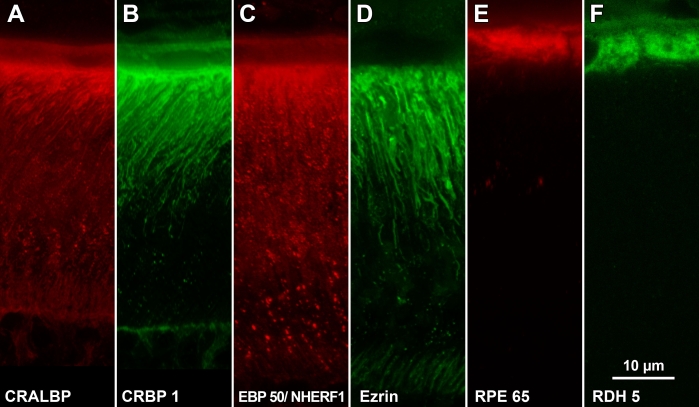
Immunostaining of albino rat retina. Sections were labeled with fluorescent probes and analyzed by laser scanning confocal microscopy. The region of outer retina in these images is similar to that of mouse retina shown in Figure 1 **A:** CRALBP is found within the whole RPE cells including the soma and apical processes (pAb UW55). **B:** The distribution of CRBP1 is similar to that of CRALBP (mAb F3). **C:** EBP50/NHERF1 is found within the soma but primarily within the apical processes (pAb B62). **D:** Ezrin is found primarily within the apical processes (pAb B64). **E:** RPE65 is found within the soma and not within the apical processes (pAb PETLET). **F:** RDH5 is found within the soma and not within the apical processes (mAb D9). Images shown are Z-projections created from LSCM image stacks (Z_t_=3 μm).

**Figure 4:**
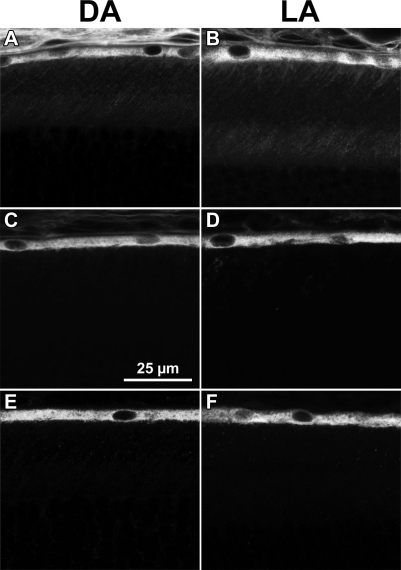
Light and dark experiments. The results illustrate the observed immunocytochemical localization of LRAT (**A** and **B**), RHD5 (**C** and **D**), and RPE65 (**E** and **F**) in retinas from dark-adapted (DA) mice (**A**, **C**, and **E**) and in retinas from mice exposed to room illumination (light-adapted, LA; **B, D, F**). Dark adapted (DA) mice were held in the dark for 16 h before sacrifice. Light adapted (LA) mice were held in the dark for 16 h, exposed to room illumination for 60 min, and sacrificed.

**Figure 5:**
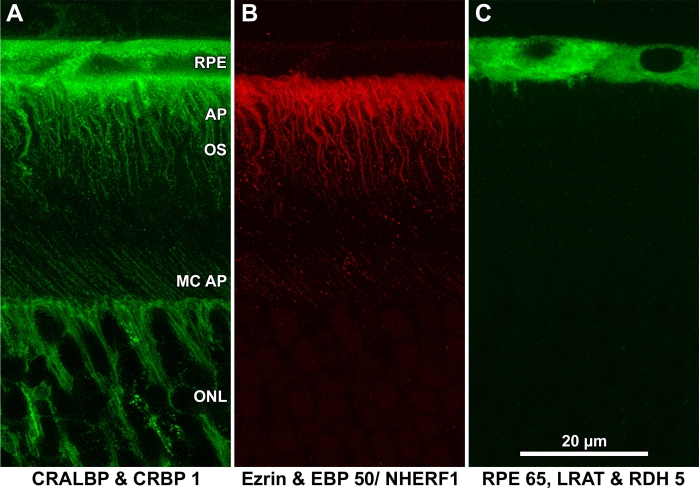
Summary of distributions in mouse retina. **A:** CRALBP and CRBP1 are present in cell bodies and apical processes of RPE cells. Anti-CRALBP is shown. **B:** Ezrin and EBP50/NHERF1 are primarily found in RPE apical processes. Anti-ezrin staining is shown. **C:** RPE65, LRAT, and RDH5 are found in RPE cell bodies. Anti-RPE54 staining is shown.

Immunolabeled mouse or rat retina sections were imaged by confocal microscopy using a Zeiss laser scanning multiphoton confocal microscope (Zeiss LSM 510 MPLSM; Carl Zeiss Microimaging, Inc., Thornwood, NY). Single plane and Z series images were collected and stored as unprocessed files. Laser scanning microscope (LSM) acquired image files, selected for publication, were pre-processed and projected, if necessary, with NIH ImageJ. JB-4 embedded sections were examined and micrographs acquired with a Nikon E1000 microscope. Adobe Photoshop version 10 (Adobe Systems, Inc., San Jose, CA) was used for final processing and figure construction. The immunofluorescence images obtained from the general area of outer retina enclosed by the box in Figure 1 are shown in Figure 2, Figure 3, Figure 4, and Figure 5.

### Preparation of tissue for immunocytochemistry

Mice or rats were euthanized by lethal injection of pentobarbital (3.6 mg/kg), their eyes removed and fixed in 4% paraformaldehyde buffered with 0.14 M sodium phosphate, pH 7.4, at room temperature (RT) for 5 min. The anterior segment of the eye was then removed, and the posterior segment (eye cup) was again immersed in the 4% paraformaldehyde fixative for 6 h at RT. The tissue was then washed with phosphate buffer containing 0.14 M sodium phosphate, pH 7.4, and 0.15 M KCl. Next, the tissue was incubated sequentially for 30 min each with 5%, 10%, and 15% sucrose buffered with 0.14 M sodium phosphate, pH 7.4, and finally in 20% buffered sucrose overnight at 4 °C. The eyecup was stored for at least 1 h 21 °C before it was embedded in a 1:1 mixture of 20% sucrose and Optimal Cutting Temperature (OCT; Ted Pella, Inc., Redding, CA) compound. Eyecups were embedded in OCT, frozen on dry ice, and cut with a cryomicrotome to produce 20 µm sections.

### Lighting environments

For the experiments depicted in Figure 2 mice and Figure 3 rats, animals were dark-adapted 16 h, euthanized, and their eyes dissected and dropped into fixative within 5 min of turning on the room illumination (165 cm below two 48” 32 W fluorescent bulbs, rated at 2800 lm each). For the light–dark experiments of Figure 4, two groups of three mice were dark-adapted overnight. Mice in a dark-adapted group were euthanized and their eyes dissected and placed in fixative in red illumination (Kodak photographic safety lamps). Mice in a light-adapted group were exposed to the room illumination described for 60 min (sufficient to produce a steady-state 50% rhodopsin bleach, determined by rhodopsin measurements; data not shown) and their eyes placed in fix in the light.

### Preparation of tissue for conventional microscopy

Mouse eyes were dissected, punctured with a 2 mm slit just posterior to the corneal limbus, and placed in 2.5% glutaraldehyde, 1.6% paraformaldehyde, 0.08 M piperazine NN-bis 2-ethanesulphonic acid (PIPES), pH 7.2, at 20 °C. After 5 min, the eyes were removed, rinsed in PBS, and the anterior segment removed. The posterior segments (eyecups) were placed in the same solution for 16 h at 5 °C and washed in PBS for 30 min. The eyes were then soaked successively for 30 min in 30, 50, 70, 95, and 100% aqueous ethanol . The dehydrated eye cups were soaked in JB-4 embedding solutions (Electron Microscopy Sciences, Hatfield, PA) for 30 min at 20 °C (2X) and for 16 h at 5 °C, and embedded in 2-hydroxyethyl methacrylate (JB-4,) using a method previously described [22]. Sections were stained in azure II and methylene blue in borax buffer, pH 10 (Richardson’s stain).

### Immunocytochemistry

Sections were blocked in 5% donkey serum, 1 mg/ml BSA, 0.3% Triton X-100 (TX-100), and PBS for 1 h at RT. Primary antibody was diluted in 0.14 M sodium phosphate, pH 7.4, 0.3% TX-100 and incubated with the sections at 4 °C for 16 h (overnight). Next, the sections were washed 3X with PBS and incubated for 2 h at RT with secondary antibody diluted in same buffer as the primary. Afterwards, the sections were mounted with 2% 1,4-diazabicyclo[2.2.2]octane (DABCO) in 90% glycerol on microscope slides. For double-labeled immunocytochemistry, sections were incubated with a mixture of the primary antibodies and later with a mixture of the secondary antibodies, as described. Nucleic acids were labeled with 4’,6-diamidino-2-phenylindole (DAPI).

## Results

### Localization of calreticulin

Calreticulin is a lectin of the endoplasmic reticulum (ER) that recognizes monoglucosylated oligosaccharide chains of nascent glycoproteins [44]. Its distribution in cells is widely used as a marker of the ER. In the outer retina, anti-calreticulin stained the cell bodies of mouse RPE cells but not their apical processes (Figure 2A).

### Localization of actin

F-phalloidin produced intense labeling of the apical processes of albino mouse RPE and Müller cells (Figure 2B). Actin was also apparent adjacent to the basal border of RPE cells and at adhering junctions between Müller and photoreceptor cells (the zonulae adherentes of the external limiting membrane).

### Localization of CRALBP

Localizations of CRALBP varied modestly between mouse and rat. In both species, CRALBP was found throughout the RPE cells, from the basal surface to the tips of the apical processes (Figure 2C and Figure 3A). In mouse retina, both somata and apical processes appeared heavily labeled (Figure 2C), whereas in rat retina a band of heavy labeling appeared in the apical processes adjacent to the somata (Figure 3A). The somata of rat RPE cells appeared lightly labeled relative to those of mouse RPE cells (compare Figure 2C to Figure 3A). Occasional patches of punctate labeling appeared over apical processes (Figure 3A). Nuclei were labeled in mouse retina but not in rat retina. CRALBP was also apparent in Müller cells in both species. We termed this localization pan-RPE.

### Localization of CRBP1

CRBP1 distribution was similar to that of CRALBP in that the protein was distributed throughout the whole RPE cells (Figure 2D and Figure 3B). Again, somata of mouse RPE were more heavily labeled than those of rat RPE (compare Figure 2D with Figure 3B). Müller cells were lightly labeled in sections from rat retina (Figure 3B). CRBP1 has also been reported in Müller cells [45,46] but in this study staining was much lighter than in RPE cells.

### Localization of EBP50/NHERF1

With anti-EBP50, fluorescence was intense over RPE processes near their origins in the cell bodies and less intense toward their tips. Lighter and more uniform labeling was also present in the cell body (Figure 2E and Figure 3C). Müller cell apical processes were lightly labeled in albino mouse and rat sections (Figure 3A). The punctate distribution observed in these two images was consistently obtained with this antibody and was especially evident in rat RPE cells. Centrifugation as well as filtration of the antibody before use did not diminish punctate staining. In addition, the collection of confocal optical sections shown began 2–3 μm below the physical surface of the section where surface aggregation phenomena would be minimized.

### Localization of ezrin

Anti-ezrin produced intense labeling of RPE apical processes in albino mouse and rat retinas (Figure 2F and Figure 3D). In contrast to the punctate appearance of EBP50/NHERF1 localization, ezrin distribution within RPE apical processes appeared much more uniform. The apical processes of Müller cells were apparent in sections of rat retina, but were labeled much less intensely compared to RPE apical processes.

### Localization of RPE65, RDH5, and LRAT

RPE65, RDH5, and LRAT, three enzymes of the visual cycle, were distributed similarly in mouse retina (Figure 2G-I). Immunofluorescence was generally restricted to the soma of RPE cells with heavier staining toward the apical side. Apical processes were not labeled. Fluorescence intensity with anti-LRAT was above background over the nucleus in the image shown (Figure 2I); however, this labeling was not always seen. A similar labeling pattern (soma but not apical processes) was observed with albino rat retina with anti-RPE65 and anti-RDH5 (Figure 3E,F, respectively). Soma, but not apical processes, were also labeled with bovine retina using a monoclonal anti-RPE65 (mAb 9–22) and with a polyclonal anti-RDH5 (results not shown). mAb 9–22 did not recognize rodent RPE65. Anti-LRAT (mAB A1) did not label RPE cells in rat retina.

### Light and dark effects

We compared the immunolocalization of LRAT, RPE65, and RDH5 in RPE from mice dark adapted for 16 h and mice exposed for 60 min to room illumination, which bleached 50% of their rhodopsin (Figure 4). No differences were noted in the localization of these enzymes.

### Summary

**Figure 6:**
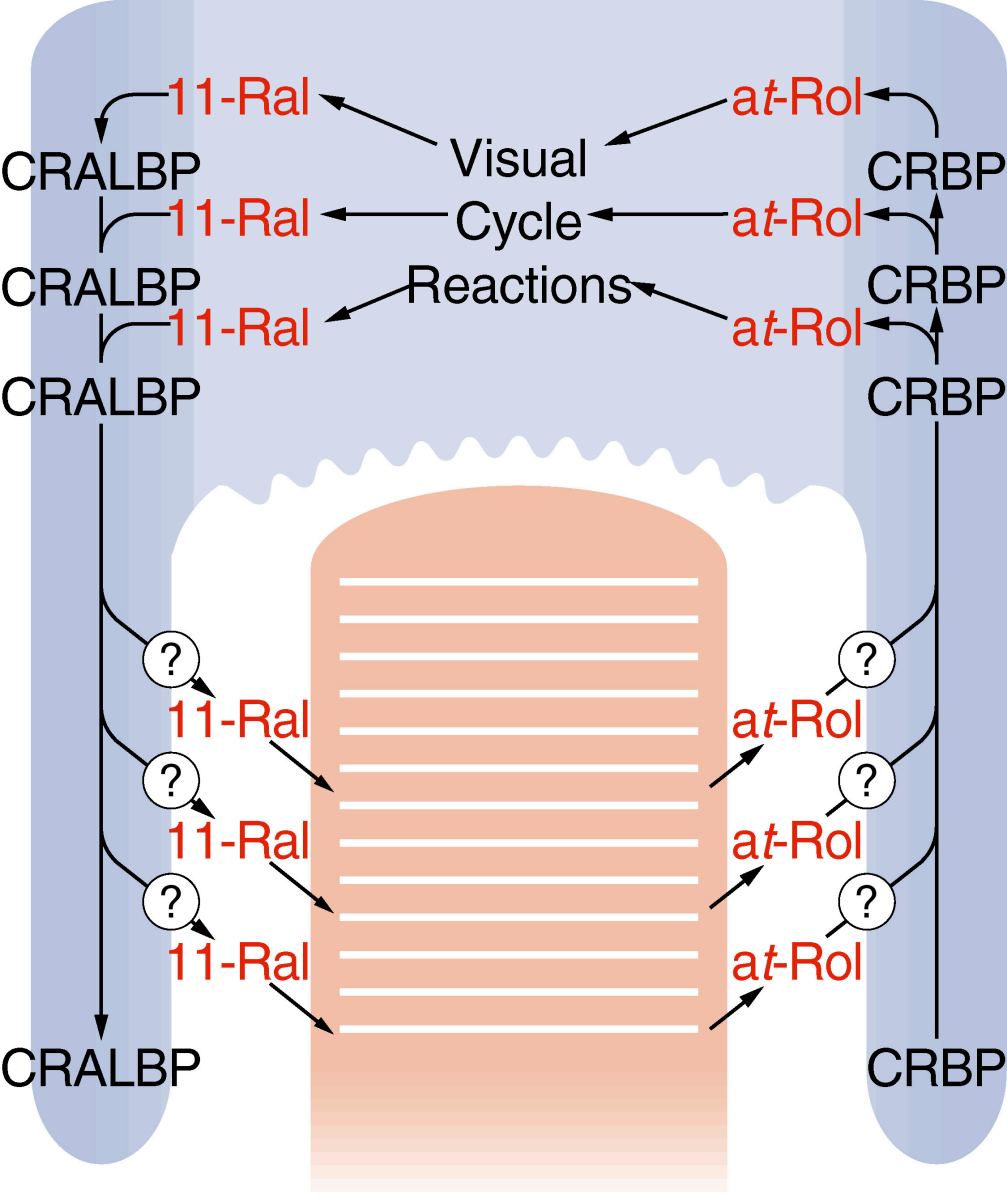
Model for visual cycle function. The different distributions reported in this study led to a model for visual cycle function. all-*trans*-Retinol, released from rod photoreceptor cells during illumination, is taken up via the apical surface of RPE cells. In turn, 11-*cis*-retinal is released from the apical surface for regeneration of rhodopsin. Visual cycle enzymes convert all-*trans*-retinol to 11-*cis*-retinal within the cell body. The presence of CRALBP and CRBP in both the cell body and apical processes allows these retinoid-binding proteins to facilitate diffusion of 11-*cis*-retinal and all-*trans*-retinol from sites of release and uptake, respectively, in the apical processes to and from enzymatic processing in the cell body. Within the apical processes, NHERF1, ezrin, and actin interact with CRALBP. Release of 11-*cis*-retinal occurs by an unknown mechanism but may be related to complex formation of interaction of CRALBP, ezrin, and EBP50/NHERF1 in the apical processes. Release and uptake of retinoids occurs along the whole apical membrane. It is depicted only from the apical processes for clarity. CRALBP and CRBP facilitate diffusion by providing binding sites for retinoids, whose diffusions are driven in either scleral or vitreal directions by concentration gradients generated by esterification of retinol by LRAT and perhaps affinity of opsin for 11-*cis*-retinal, respectively.

The localization of LRAT, RPE65, and RDH5, the three visual cycle components we observed in this study, are summarized in Figure 5. A model outlining the functions of these components is shown in Figure 6 and outlined in the Discussion.

## Discussion

In this study, we determined the localization of three enzymes of the rod visual cycle in albino rodent RPE and compared these to the known localization of other components of the rod visual cycle. The striking finding that emerged is that localizations fell into three distinct patterns: somata only, apical processes only (or mainly), and pan-RPE. Restriction of the enzymatic reactions to the somata could provide concentration gradients to drive diffusion of the all-*trans*-retinol toward the somata and 11-*cis*-retinal toward the apical processes. The pan-RPE localizations of CRALBP and CRBP1 reinforce their proposed roles in mediating diffusion of retinoids between the apical membranes, where release and uptake of retinoids must occur, and the somata where the visual cycle reactions take place. Finally, components largely confined to the RPE apical processes are well placed to mediate release of 11-*cis*-retinal from CRALBP and ultimately from RPE.

### Actin

Numerous investigators have localized f-actin to RPE apical processes [47,48] and other regions of the RPE cell. Fluorescence was particularly intense over both RPE and Müller cell apical processes and along the basal border of RPE cells. The presence of actin in these processes provides a rationale for localization of ezrin, an actin-binding protein. Others have noted ezrin localization near the basal side of RPE cells.

### CRALBP

A pan-RPE distribution of CRALBP was suggested in the first published immunocytochemical studies with bovine and primate retinas in which somata were heavily labeled by anti-CRALBP and apical processes appeared as a fluorescent fringe [40]. Immunogold electron microscopy revealed a uniform distribution of CRALBP in the cytoplasm of squirrel [49] and bovine [50] RPE cells. Subsequent studies of retinas with conventional [22] and laser scanning confocal microscopy [34] confirmed this pan-RPE localization. The only exception to this observation is the rat retina shown Figure 3 where the somata appeared less heavily labeled than the apical processes. CRALBP was also noted to outline the profile of Müller cells, including their apical processes [40].

### CRBP1

CRBP1 was localized to RPE and Müller cells of rat and bovine retinas [45,46], and its presence in RPE apical processes was noted [45,51]. The pan-RPE distribution was particularly evident in the images we obtained with confocal microscopy. CRBPI was also observed in Müller cells of rat retina [45]. In our studies, Müller cells were less heavily labeled with anti-CRBP1 than RPE cells.

### Ezrin

Ezrin displayed a striking localization to apical processes of RPE, as noted in previous studies [52]. Labeling of apical processes of Müller cells was much lighter and not always apparent unless the fluorescence intensity of RPE cells was allowed to saturate. The apical localization of ezrin likely has its origin in the affinity of ezrin for filamentous actin, which is found within the RPE apical processes. Ezrin has an actin-binding domain at its C-terminus. A compound FERM (Band 4.1, ezrin, radixin, and moesin) domain at its N-terminus has an affinity for EBP50/NHERF1, a scaffold protein with two PDZ domains [36,37].

### EBP50/NHERF1

EBP50/NHERF1 was clearly evident within the apical processes of rat and mouse RPE cells and also evident within the somata, where it had been noted previously [52]. In rat RPE cells, EBP50/NHERF1 appeared in a band near the basal surface. The punctate character of EBP50/NHERF1 staining in Müller and RPE apical processes was particularly striking and was present regardless of efforts to minimize nonspecific aggregation. A punctate distribution of moesin, a related protein, was noted in retina [53]. Recent studies demonstrated that CRALBP has a PDZ motif at its C-terminal sequence (N/DTAL/F) and in vitro binds to at least one of the PDZ-domains of EBP50/NHERF1 [34,35]. As we have mentioned, it also has affinity for ezrin. It is possible that the punctate foci represent complexes of ezrin, EBP50/NHERF1, CRALBP, and perhaps actin. However, we have no evidence to support this contention at present.

### LRAT

LRAT was localized to RPE cells in previous studies, but it was unclear from published images if the protein was expressed in RPE apical processes [43]. Our results with albino mouse retina indicate that the enzyme is expressed in the cell bodies of RPE cells but not in the long apical processes. Expression was not observed in inner retina in our studies.

### RPE65

RPE65 was originally identified as p63, a putative membrane receptor for retinol-binding protein [54]. Immunocytochemistry with anti-p63 and bovine retina revealed relatively exclusive labeling in the RPE layer of bovine retina with apparent labeling of apical processes [55]. However, subsequent immunocytochemical localization with confocal microscopy and the same antibody did not show evidence of labeled apical processes [56]. Our results demonstrate that the enzyme is in the somata and not in the long apical processes.

### 11-cis-retinol dehydrogenase

Anti-RDH5 labeled RPE somata heavily. Long apical processes were not labeled. RDH4, the mouse ortholog of RDH5, was previously localized to the RPE layer of mouse in the initial report of its expression [57]. RDH11, another dehydrogenase with *cis*-retinol dehydrogenase activity, was previously localized to the RPE layer of monkey and bovine retina [42]. Apical processes do not appear to be labeled in these studies; however, fine anatomic localization was not the primary aim of either study.

### Endoplasmic reticulum

The known enzymes of the rod visual cycle are membrane-associated as noted in studies of the isomerohydrolase (RPE65) [58,59], LRAT [9,11,12,60], and *cis*-RDH [61,62]. Membranes bearing these enzymes have not been extensively characterized; however, it is likely that they are derived from the ER. The restriction of these enzymes to RPE somata suggests that the ER doesn’t extend into the apical processes. Restriction of calreticulin to RPE somata is consistent with this suggestion. Calreticulin is an ER-resident lectin involved in the quality control of nascent glycoproteins [44]. However, tubular structures are evident in the apical processes in some [63,64] but not all [65-67] high-resolution electron microscopic studies. The mechanism for restriction of enzymes of the visual cycle to RPE somata awaits more extensive studies.

### Light and dark effects

The exposure to light for 60 min did not affect the distribution LRAT, RPE65, or RDH5, relative to the dark-adapted retina (Figure 4), nor were there major changes in the distributions of CRALBP and CRBP1 (results not shown). Determining whether there was a quantitative change in the punctate character of EBP50/NHERF1 label will require a more extensive study.

### Model

The distribution of visual cycle components documented in this study led to a model for compartmentalization of reactions and processes of the visual cycle within RPE cells (Figure 6). During active cycling of visual pigment, the anatomy of the outer retina dictates that both release of 11-*cis*-retinal and uptake of all-*trans*-retinol must occur via the RPE apical plasma membrane. Based on our localization of LRAT, RPE65, and RDH5, transformation of all-*trans*-retinol into 11-*cis*-retinal must occur with the somata of the RPE cells. The two retinoid-binding proteins CRALBP and CRBP1, which are found throughout the RPE cells, overlap these two locations, allowing the binding proteins to facilitate transport of retinoids between the cell bodies and apical membranes.

We do not suggest that CRALBP and CRBP1 undergo massive displacements within the RPE cell with their retinoid cargos. It is more likely that the proteins provide binding sites for the retinoids, which then diffuse through the aqueous phase via multiple cycles of binding and dissociation. A concentration gradient to drive sclerad diffusion of all-*trans*-retinol would be generated by the powerful LRAT activity in the cell body [11,12], which would immediately esterify the vitamin and reduce its concentration. Similarly, multiple cycles of binding and dissociation of 11-*cis*-retinal and CRALBP would facilitate the diffusion of this retinoid from the site of its production in the cell body to sites of its release in apical membranes. The driving force for vitreal diffusion of 11-*cis*-retinal is unknown but could be its release from CRALBP as well as from the RPE cell, and ultimately conjugation with opsin.

There is ample published evidence that CRALBP can be loaded with 11-*cis*-retinal by enzymes of the visual cycle. Apo-CRALBP stimulated the rate of 11-*cis*-retinol formation by isomerohydrolase [23,24] and is routinely added to in vitro assays of this enzyme [23,24]. RPE microsomes catalyzed the oxidation of 11-*cis*-retinol bound to CRALBP by NAD(P) and the product of the reaction, 11-*cis*-retinal, was found associated with the binding protein [68,69]. Analysis of visual cycle retinoids in *Rlbp1^−/−^* mice demonstrated that the flow of retinoids was impaired at the isomerization reaction, consistent with the proposed role of CRALBP as an acceptor of 11-*cis*-retinol [22]. Humans with mutations in the CRALBP gene (*RLBP1*) suffer from delayed dark adaptation [21], demonstrating an impairment of the visual cycle.

The role of CRBP1 as a carrier of all-*trans*-retinol for esterification by LRAT has been well established in other tissues [27,28,30], and analysis of *Rbp1^−/−^* mice revealed decreased stores of retinyl ester in liver [25]. Analysis of visual cycle retinoids in *Rbp1^−/−^* mice during recovery from a flash revealed a decrease in retinyl esters and an increase in all-*trans*-retinol, consistent with the role for the protein as a substrate carrier for esterification of all-*trans*-retinol [26].

### Release of 11-cis-retinal from CRALBP

The model for the rod visual cycle proposed here demands a mechanism for release of 11-*cis*-retinal from CRALBP and from the apical membrane of the RPE cell. However, 11-*cis*-retinal is tightly bound to CRALBP (K_d_ approximately 15 nM) and its functional group is sequestered from water-soluble carbonyl reagents [68,69]. To date, no high-affinity acceptor for 11-*cis*-retinal has been found. The presence of CRALBP, ezrin, and EBP50/NHERF1 in RPE apical processes suggests that they could interact to form a multiprotein complex, which might be transient given the potential for regulation of ezrin reactivity [36]. Complexes of CRALBP and EBP50/NHERF1 have been demonstrated in vitro [34,35]. The functional significance of these multiprotein complexes is unknown at present. However, they could increase the residence time of CRALBP in the vicinity of the apical plasma membrane where it could come in contact with acidic glycerophospholipids of the cytoplasmic leaflet [70]. We have recently demonstrated that acidic glycerophospholipids, especially phosphatidic acid and phosphatidylserine, release 11-*cis*-retinal from CRALBP [71].
